# From the president’s desk

**DOI:** 10.4102/safp.v63i1.5287

**Published:** 2021-03-09

**Authors:** Robert Mash

**Affiliations:** 1 Division of Family Medicine and Primary Care, Faculty of Medicine and Health Sciences, Stellenbosch University, Cape Town, South Africa; 2 South African Academy of Family Physicians, Cape Town, South Africa

The South African Academy of Family Physicians has a new council (Table 1) for the next three years (2020-2023). The council is scheduled to meet in February to set the strategic direction for the academy in a context of huge uncertainty with regard to the coronavirus pandemic and roll-out of vaccinations. In this editorial, I outline some of the initiatives that we plan to focus on in the next year.

**TABLE 1:** The Academy Council 2020–2023.

Name	Organisation
Tasleem Ras (Director)	University of Cape Town
Bob Mash (President)	Stellenbosch University
Hanneke Brits (Treasurer)	University of the Free State
Nonhlanhla Khumalo (Director)	University of the Witwatersrand
Indiran Govender	University of Pretoria
Nnanile Nyalunga	Sefako Makgatho Health Sciences University
Sizeka Maweya	University of Limpopo
Andrew Ross (Vice-President)	University of KwaZulu-Natal
Olukayode Adeleke	Walter Sisulu University
Wim Beukes	Private practice
Jenny Nash (Secretary)	Rural Doctors Association
Biano Hobson	Private practice
Chantelle van der Bijl	Trainee

At our Annual General Meeting there was support for the introduction of a new category of membership for medical students. We hope to encourage students to take an interest in the discipline of family medicine even before they qualify and to hopefully form interest groups in family medicine and primary health care at their universities. In this way we can begin to build the pipeline for future family doctors at an earlier stage. This year also sees the introduction of the new 6-month internship rotation in family medicine and primary care for second-year interns. This is also a huge opportunity to attract and retain more doctors in the field of family medicine. For all our members, the academy is offering a set of branded scrubs so that they can identify themselves and promote our discipline in the workplace (visit our on-line shop to order at https://saafp.org).

The academy will continue to advocate for the discipline with the National Department of Health in relation to policy for district health services, human resources for health and national health insurance. In addition, we will continue to engage with the private sector to ensure that family physicians are appropriately recognised and remunerated. The academy has a number of strategic partners, including the South African Medical Association, Rural Doctors Association of South Africa, College of Family Physicians, World Organization of Family Doctors and Physicians for Planetary Health.

The *South African Family Practice Journal* encourages more continuing professional development (CPD) articles written by family physicians for all doctors working in the field of family medicine and primary care. These articles will focus not only on clinical competencies but also on the important areas of capacity building, clinical training, clinical governance and leadership. The journal has also put out a call for our members to submit artwork that can be used on the front cover of the journal. In 2021, we hope to accelerate our offering of online CPD short courses and to position ourselves in relation to the new guidelines being discussed by the Health Professions Council of South Africa.

Our national Education and Training Committee will be chaired for the next 3 years by Prof. Hanneke Brits. They are busy revising the national programmatic learning outcomes for the training of family physicians. The biggest change is in relation to community-orientated primary care, and in this edition of the journal the ideas behind this are explained in a CPD article. We also hope to continue our training of clinical trainers for the workplace and to move forward with a national electronic portfolio of learning and workplace-based assessment.

Last year, our National Family Practitioners Conference was postponed to August 2021. Given the many uncertainties with the coronavirus pandemic, we plan to hold this conference virtually with the help of technology from an experienced conference organiser. This will also make it easier and cheaper for many people in South Africa and other countries to attend. Please diarise the provisional dates of 13–14 August 2021.

Finally, it is my sad duty to report that the discipline has lost two senior figures, both from KwaZulu-Natal. Professor Cyril Naidoo was the previous Head of Department and instrumental in the recognition of family medicine as a speciality in 2007. Professor Papoo Cassimjee was a pioneer of family medicine and founded the Department of Family Medicine at the University of KwaZulu-Natal. You will find more extensive tributes to both leaders of family medicine on the South African Family practice website (https://saafp.org/2021/01/18/obituary-professor-cyril_naidoo/; https://saafp.org/2021/01/14/obituary-prof-cassimjee/).

